# Genito-Urinary Surgery

**Published:** 1905-08-19

**Authors:** 


### GENITO-URINARY SURGERY.

(*Continued from page* 343.)

**Conservative Operations for Hydronephrosis.**—F. Gardner[6] points out that cases of hydronephrosis not curable by nephropexy or by the external liberation of the ureter are due to a vicious implantation of the ureter into the renal pelvis, either because it is not implanted at the most dependent part, or it presents a stricture, or is bent or twisted or compressed by an abnormal renal artery. The methods of treatment available for these conditions are :—(1) Division of the spur when the ureter is implanted too high in the pelvis; (2) uretero-pyeloplasty in which the orifice of communication with the pelvis is divided longitudinally and sutured transversely; (3) uretero-pyeloneostomy, in which the ureter is divided and the end implanted into the lowest part of the pelvis; (4) pyeloplication, in which the size of the pelvis is diminished by infolding; (5) orthopædic resection, in which the greater part of the dilated pelvis and kidney is resected; (6) lateral anastomosis of the ureter; and (7) nephro-cysto-anastomosis, in which the dilated renal pelvis is directly anastomosed with the bladder. If infection is present (pyonephrosis) a preliminary nephrostomy is essential.

**Renal Calculus.**—G. E. Brewer, New York,[7] recently opened a discussion on renal surgery at the New York Academy of Medicine. He alluded to 36 cases of renal trouble in which the presence of calculus was strongly suspected. In only 21 of these did subsequent operation reveal a stone. Of the remaining 15, nine showed other lesions, but in six no lesion could be found. Thus the diagnosis of stone in the kidney was wrongly made in 41 per cent. of the cases. Pain was the most constant symptom of calculus. It was either paroxysmal or constant, and in some mild, in others severe. Of cases with typical renal colic or constant pain not due to stone one had intermittent hydronephrosis, two had chronic interstitial nephritis, one had multiple abscesses, four had no discoverable lesion, and two had suppurative disease of the kidney. Frequent or painful micturition was present in more than half the cases of stone, and tenderness was almost constantly present. Pyuria was present in 16 of the 21 cases, the kidney was palpable in a third of the cases. The writer considers $x$-ray examination to be the most valuable method of examination for the diagnosis of calculous disease of the kidney or ureter. In three cases shadows were given with the $x$-rays and yet no stones were found. In one of these the shadow was due to a calcareous appendix epiploica adherent to the peritoneum over the ureter. Possible sources of error in $x$-ray plates are shadows due to calcified lymph nodes, phleboliths, calcareous masses in the sacro-sciatic ligament, and calcified appendices epiploicæ. Dr. Cole recommends the rejection of all plates which do not show the outline of the psoas muscle and the transverse processes of the lumbar vertebræ, and that all shadows which have not well-defined edges he regarded with suspicion. That calculus often exists without pain is evidenced by the statement of Bruce Clark, who reported 24 autopsies upon calculous patients, in 13 of which there had been no subjective symptoms during life. H. A. Fowler,[8] discussing the diagnosis of stone, alludes to the importance of taking a full and correct history. The patient never remembers to mention all his symptoms and valuable information is missed if the history is taken hurriedly. With respect to the state of the urine the cases fall into two groups—namely, (1) The infected, with pus in the urine; and (2) the non-infected. The urine in the latter cases may be absolutely normal. The cystoscope gives valuable information on the following points: (1) Presence or absence of vesical involvement—calculus, tumour, etc.; (2) difference in the two sides of the trigone, suggesting a lesion higher up, either calculous or tuberculous; (3) presence of ureteral calculus at the mouth of the ureter; and (4) in cases of pyuria or hæmaturia of renal origin the side of the disease may be recognised, if unilateral.

**Tuberculosis of the Kidney.**—Leopold Casper (Berlin),[9] has discussed the diagnosis and treatment of this condition. He states that tuberculosis of the kidney is not an ascending infection but is due to infection through the blood-stream, and that tuberculosis of the genital tract must be dissociated from it. Usually one kidney only is affected, it being only in the late stages that the second kidney is attacked. The disease is commoner than has been thought and occurs much more often than it is diagnosed. Painful and frequent micturition and pain in the kidney are symptoms of little diagnostic value, for they are met with in almost any disease of the kidney. True attacks of renal colic are often present, and are due to the passage of caseous masses down the ureter. In the early stages the patients often appear to be in perfect health. The most important objective symptom is the altered condition of the urine. In a few cases the earliest sign is an attack of hæmaturia. Pus is almost always present in the urine and blood, albumen, casts and epithelial cells can frequently be found Tubercle bacilli can be demonstrated in about 80 per cent. of the cases. Tuberculous urine, even with pus present, is often sterile. Similarly sterile pleural effusions are nearly always tubercular. Convincing, but lengthy, is the diagnosis by the injection into guinea-pigs of the centrifugalised sediment of the urine. The animals must first be tested with tuberculin to discover if they are free from tuberculosis. The reaction of a patient to the tuberculin test is not reliable, for in the majority of men small tuberculous nodules may be present in various parts and yield a reaction. Also an injection of tuberculin may light up the disease and render it dangerous to life. Küster and others, however, deny that the presence of tubercle bacilli in the urine proves that the patient has urinary tuberculosis. The bacilli may be in some distant organ and may be merely shed in the urine. This would not apply if the bacilli were present only in the urine of one ureter. Primary tuberculosis of the bladder is rare. The presence of an enlarged kidney on one side may lead to an error in diagnosis, because sometimes the diseased kidney is shrunken and the sound one enlarged from compensatory hypertrophy. The author has found that the prognosis of operable cases is much worse if they are not operated upon than if they are. Therefore all cases should be operated upon as soon as the diagnosis is established. The most important point to decide before operating is the sufficiency of the other kidney. He gives an interesting statistical table which shows that those surgeons who use the modern methods of ascertaining the sufficiency of the opposite kidney get much better results than those who do not use these methods.

[6] Vide Abst. Med. Chron., April 1905, p. 33. [7] Med. Rec., Feb. 18, 1905, p. 243. [8] Ibid., Feb. 4, 1905, p. 171. [9] Vide Abst. Med. Chron., March 1905, p. 394.

